# Supplementary material for: Three Molecular Modification Strategies to Improve the Thermostability of Xylanase XynA from Streptomyces rameus L2001
Source: Foods. 2023 Feb 18;12(4):879. doi: 10.3390/foods12040879 (PMC9957083; doi:10.3390/foods12040879)
Supplement: Supplementary file 1 [file foods-12-00879-s001.zip › foods-2198984-supplementary.pdf]

Supplementary

Table S1. The primers used in this work.

Primers	Sequences (5’ - 3’)
Q24A-F	CTCGTTCTGGACCGACGCG**GCG**GGCAC
Q24A-R	GGTCATCGAGACCGTGCC**CGC**CG
K104A-F	CGGCCCACGGGGACGTAC**GCG**GGCAG
K104A-R	CACTGGTGACACTGCC**CGC**GTAC
K143A-F	CGTGCGGCAGTCG**GCG**CGCACCGG
K143A-R	GGTGCCGCCGGTGCG**CGC**CGACTGC
V3C-F	GATATACCATGGCCACG**TGC**GTCAC
V3C-R	GGTTCGTGGTGAC**GCA**CGTGG
T30C-F	GCACGGTCTCGATG**TGC**CTGG
T30C-R	CCAG**GCA**CATCGAGACCGTGC
N36C-F	TGGC**TGC**TACAGCACCAGCTGGCG
N36C-R	CTGTA**GCA**GCCACCGGAGCCCAG
T188C-F	CAGCGGCAACTCCAATATC**TGC**GTGT
T188C-R	GACAC**GCA**GATATTGGAGTTGCCGCTG
S109C-F	GGCAGTGTCACC**TGC**GACG
S109C-R	CGTC**GCA**GGTGACACTGCC
N153C-F	CACCATCACCACCGGC**TGC**CACTT
N153C-R	TG**GCA**GCCGGTGGTGATGGTG

The mutated sites are marked red and bold.

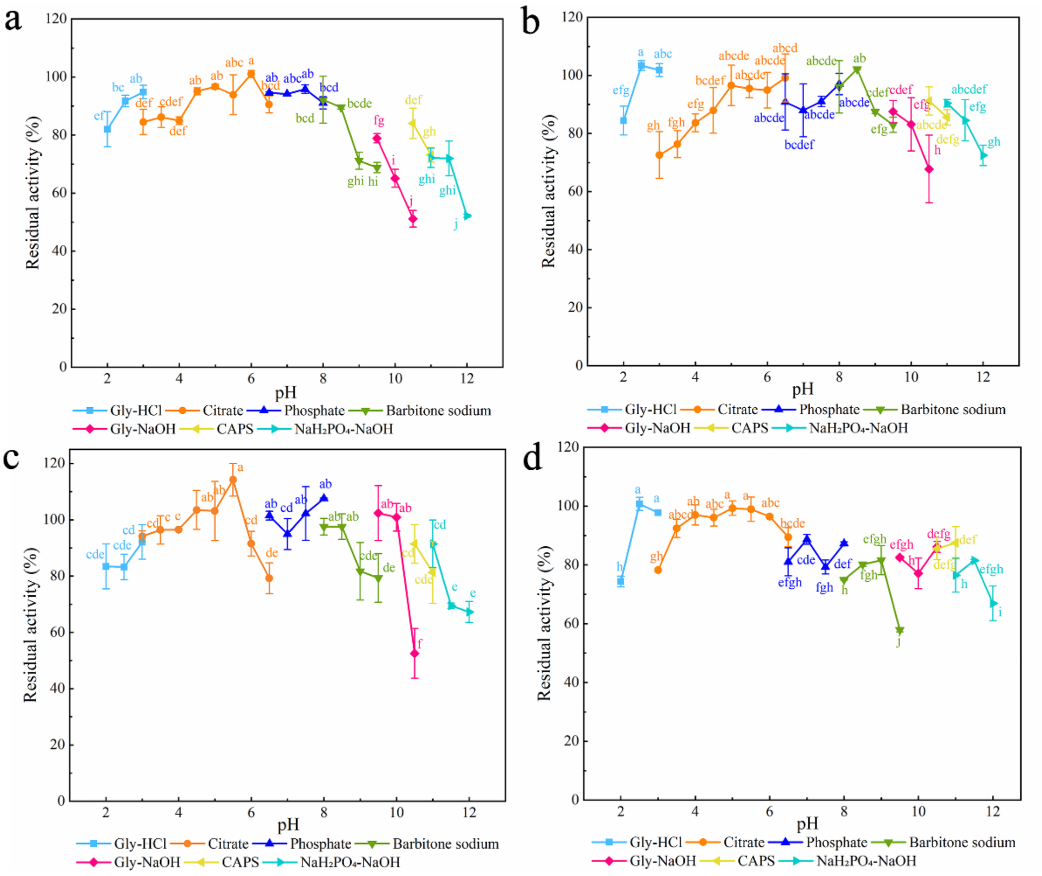

Figure S1. The pH stability of SER mutated enzyme and XynA. (a) Q24A; (b) K104A; (c) K143A; (d) XynA. pH stability was determined by incubating at varying pH from 2.0 to 12.0 at 37°C for 30 min, the activity of untreated xylanase was defined as 100%. Using beechwood xylan as substrate, buffers used were 50.00 mM Gly-HCl buffer (2.0–3.0), citrate buffer (pH 3.0–6.5), phosphate buffer (pH 6.5–8.0), barbital sodium buffer (pH 8.0–9.5), Gly-NaOH buffer (pH 9.5–10.5), CAPS buffer (pH 10.5–11.0), NaH_2PO_4-NaOH buffer (pH 11.0–12.0). Means within rows followed by the same letter were not significantly different ($p < 0.05$).

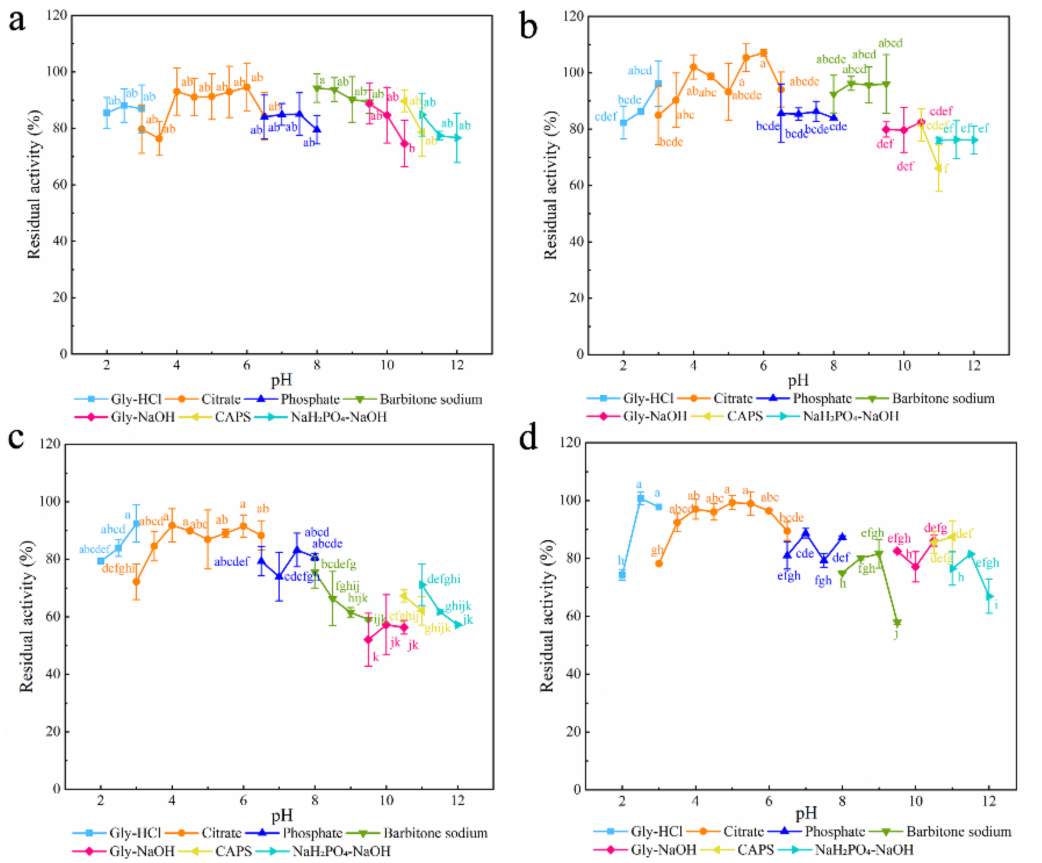

Figure S2. The pH stability of disulfide mutant enzyme and XynA. (a) XynA-VT; (b) XynA-NT; (c) XynA-SN; (d) XynA. pH stability was determined by incubating at varying pH from 2.0 to 12.0 at 37°C for 30 min, the activity of untreated xylanase was defined as 100%. Using beechwood xylan as substrate, buffers used were 50.00 mM Gly-HCl buffer (2.0–3.0), citrate buffer (pH 3.0–6.5), phosphate buffer (pH 6.5–8.0), barbital sodium buffer (pH 8.0–9.5), Gly-NaOH buffer (pH 9.5–10.5), CAPS buffer (pH 10.5–11.0), NaH_2PO_4-NaOH buffer (pH 11.0–12.0). Means within rows followed by the same letter were not significantly different ($p < 0.05$).
